# Restriction Enzyme Body Doubles and PCR Cloning: On the General Use of Type IIS Restriction Enzymes for Cloning

**DOI:** 10.1371/journal.pone.0090896

**Published:** 2014-03-11

**Authors:** Eszter Tóth, Krisztina Huszár, Petra Bencsura, Péter István Kulcsár, Barbara Vodicska, Antal Nyeste, Zsombor Welker, Szilvia Tóth, Ervin Welker

**Affiliations:** 1 Institute of Biochemistry, Biological Research Centre, Hungarian Academy of Sciences, Szeged, Hungary; 2 Institute of Molecular Pharmacology, Research Centre for Natural Sciences, Hungarian Academy of Sciences, Budapest, Hungary; New England Biolabs, Inc., United States of America

## Abstract

The procedure described here allows the cloning of PCR fragments containing a recognition site of the restriction endonuclease (Type IIP) used for cloning in the sequence of the insert. A Type IIS endonuclease - a Body Double of the Type IIP enzyme - is used to generate the same protruding palindrome. Thus, the insert can be cloned to the Type IIP site of the vector without digesting the PCR product with the same Type IIP enzyme. We achieve this by incorporating the recognition site of a Type IIS restriction enzyme that cleaves the DNA outside of its recognition site in the PCR primer in such a way that the cutting positions straddle the desired overhang sequence. Digestion of the PCR product by the Body Double generates the required overhang. Hitherto the use of Type IIS restriction enzymes in cloning reactions has only been used for special applications, the approach presented here makes Type IIS enzymes as useful as Type IIP enzymes for general cloning purposes. To assist in finding Body Double enzymes, we summarised the available Type IIS enzymes which are potentially useful for Body Double cloning and created an online program (http://group.szbk.u-szeged.hu/welkergr/body_double/index.html) for the selection of suitable Body Double enzymes and the design of the appropriate primers.

## Introduction

Although ligase-free cloning methods are widely used [1], [2], [3], restriction digestion and ligation will most probably remain as one of the main approaches for cloning/subcloning DNA (deoxyribonucleic acid) fragments. By flanking a PCR (polymerase chain reaction) primer with the recognition sites of the appropriate restriction endonucleases (ENase), a DNA fragment can be cloned to virtually any cloning sites that are used to linearize the acceptor vector. The only frequently occurring obstacle arises when the DNA fragment to be cloned contains the recognition site(s) of the enzyme(s) to be used for cloning. Here we demonstrate a strategy that effectively bypasses this problem by using a Body Double (BD) for the particular enzyme: a Type IIS ENase. This strategy also offers solutions for miscellaneous cases where the Type IIP restriction site used for cloning should not be present in the product vector as it is required for further cloning steps or for background clearing.

Type IIP enzymes [4] that generate identical, palindrome overhangs are commonly used in cloning experiments since the protruding ends of the two DNA fragments generated by these enzymes are generally compatible for joining by a DNA ligase. In contrast, Type IIS enzymes [4], [5], [6] are less frequently employed since they generate overhangs with arbitrary sequences which are not complementary, and thus, are not suitable for joining. Nevertheless, their unique features have given rise to several genetic engineering applications as detailed below.

In principle, a wide variety of overhangs can be produced by a Type IIS restriction enzyme (*e.g.* 256 different, typically non-palindromic, overhangs can be generated by employing a Type IIS restriction ENase that produces a four-nucleotide overhang). These non-palindromic overhangs permit the insertion of a DNA fragment into a plasmid in only one orientation when a single Type IIS enzyme is used to linearize the plasmid (whereas when a single Type IIP enzyme is used to linearize the vector, the DNA fragment is usually inserted in both orientations). It also permits the assembly of several DNA fragments in a unique order and orientation employing a single ligation reaction [7], [8], [9], [10], [11].

The ability of a Type IIS enzyme to cut outside its recognition sequence has been explored for cloning of fragments with seamless junctions [12], [13], [14], [15], [16], for precise trimming of DNA molecules [17], [18], [19], [20], [21], for precise retrieval of a DNA fragment [summarized in [5]] and for constructing universal restriction enzymes [22], [23] [for reviews [5], [24], [25]]. Furthermore, by properly orientating the recognition and the cleavage sites, two digested fragments can be ligated to generate a product in which the original restriction site is absent. This property has been used to replace the two steps of digestion and ligation by a single restriction-ligation step [26] and to design a strategy for sequential assembly of pre-made modules [27]. These various properties of Type IIS restriction enzymes have also been used to design a method that allows subcloning of a DNA fragment from an entry clone into an expression construct with almost 100% efficiency in a single step and tube, without adding any additional nucleotide sequences to the final cloned product [28]. The ability of Type IIS enzymes to cut arbitrary sequences outside their recognition site has been exploited to generate palindromic overhangs on a vector that are compatible with a Type IIP enzyme. This has allowed the cloning of an insert digested with BamHI site into a vector that possess several BamHI sites [29]. Since this approach requires the construction a new vector for each Type IIS ENase needed for cloning such a method has not gained widespread use. Here we use the same approach to generate overhangs on a PCR fragment that offer considerably more practical benefits.

## Materials and Methods

Pfu (*Pyrococcus furiosus*) polymerase, dNTP (deoxyribonucleotide), T4 DNA ligase, and DNA ladder were purchased from Thermo Scientific. Restriction enzymes were purchased from Thermo Scientific and New England Biolabs. NaCl was purchased from Reanal, Tripton from Fluka, Bacto-agar from Biolab, and Agarose from Lonza.

The synthesis of the oligonucleotides used and the sequencing of the DNA constructs were carried out by Microsynth AG (http://microsynth.ch/).

### 1. Plasmid construction

Plasmids were constructed by PCR amplification of the insert, restriction digestion and ligation using standard molecular biology methods, briefly: the host vectors [pEGFP-C1 (Clonetech), pTXB3 (New England Biolabs), pTRCHisA (Invitrogen), psiSTRIKE-_hM_GFP (Promega), pACS [30], pSB [31]] were purified with Qiagen miniprep kit and digested by restriction enzymes (RE, REP: Type IIP restriction enzyme, RES: Type IIS restriction enzyme). The linearized vectors were purified from agarose gel using a NucleoSpin DNA purification kit (Macherey-Nagel) and the concentration of the samples was estimated on an agarose gel stained with ethidium bromide.

The inserts were generated by PCR using Pfu polymerase, the sequences of the primer-pairs used and the conditions of the PCR reactions are listed in Supplementary Table S1. The amplified DNA fragments were purified from agarose gels using NucleoSpin DNA purification kits and digested by a Type IIP ENase and/or a BD enzyme of the Type IIP ENase that was used to linearize the acceptor vector.

Enzymatic reactions in the case of the i) insert: 100–3000 ng purified PCR product was digested by appropriate amount of enzyme and 4 µl of 10× reaction buffer in 40 µl of final volume for O/N (overnight) at the appropriate temperature; ii) vector: 2000–4000 ng plasmid DNA was digested by appropriate amount of enzyme and 2 µl of 10× reaction buffer in 20 µl of final volume for 4 hours at the appropriate temperature.

When necessary the digested fragments were purified again and the concentrations of the inserts were estimated on agarose gels, as described above. A 1∶3 vector∶insert molar ratio was used for the ligation reactions.

After inactivating the ligase by heating to 65°C for 15 minutes, background coming from the undigested and/or the re-circularized acceptor vectors was reduced by using an appropriate restriction enzyme whenever it was possible (i. e. the acceptor vector contained its recognition sequence and the product vector did not).

Chemically competent DH5α *Escherichia coli* bacteria were transformed with the products of the ligation reactions and were grown on Luria Bertrani (LB) agar plates containing the required antibiotics (ampicillin (Sigma) or kanamycin (Sigma)). A day later single colonies were picked from the plate, inoculated into and grown overnight in LB medium containing the appropriate antibiotics. Plasmids were purified from the overnight cultures as above and tested by restriction mapping for the presence of the insert. Selected clones were sequenced by Sanger sequencing (Microsynth AG).

### 2. Online Body double finder program

The Body Double enzyme database was generated by manually selecting those Type IIS or Type IIP ENases that cleave arbitrary sequences outside from their recognition sites in a predictable manner and whose digested product can be effectively religated.

Our program analyses the insert sequence input by the user and lists possible (non-cutting) Body Double enzymes (usually Type IIS enzymes) that may produce the given Type IIP overhang. The Body Double enzymes are listed on such a way that more suitable ones (i.e.: those with shorter primers) first (appear at the top of the list). The program designs the primer sequences, which contain the given Type IIS recognition site in one of two orientations; each is appropriate to generate the desired overhangs with or without incorporating the Body Double enzyme to the product vector. The 5′ end of the tail also contains a few (1 to 6) flanking base pairs to ensure efficient cleavage by the enzyme.

The webpage was written using the Javascript program language.

## Results

### 1. Using Body Double restriction enzymes to facilitate cloning when the PCR fragment contains an internal site(s) of the Type IIP restriction enzyme to be used

**Figure 1:**
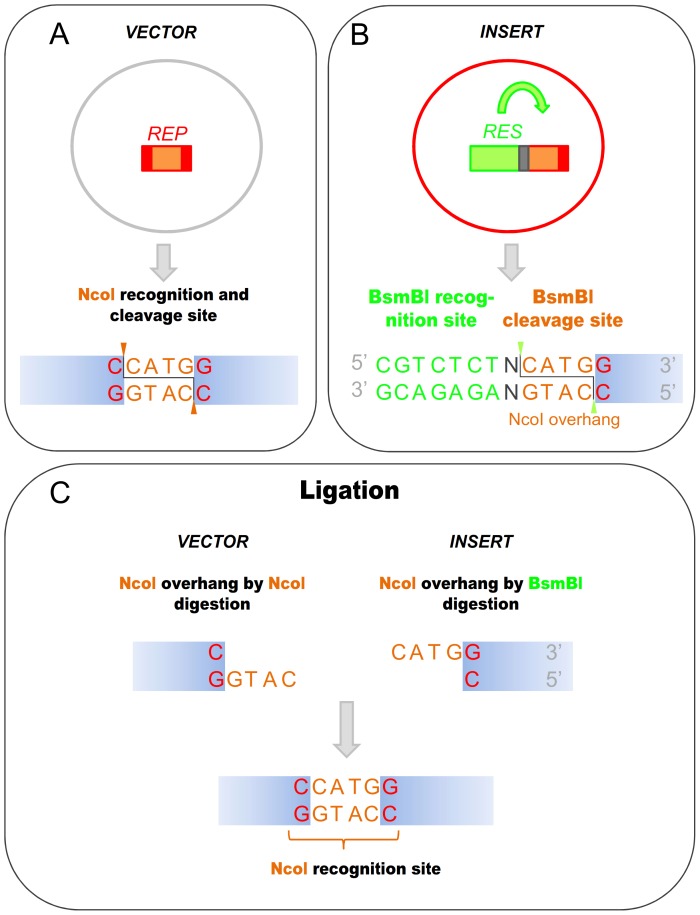
Ligation of NcoI overhangs generated by NcoI and its Body Double reconstitutes the NcoI recognition site. **A.**) Sequence of NcoI recognition and cleavage site **B.**) Sequence of BsmBI recognition and cleavage site, the latter corresponding to an NcoI overhang **C.**) Ligation of the NcoI overhangs generated by different restriction enzymes reconstitutes the NcoI site. REP: Type IIP restriction enzymes, their recognition sites are represented by red boxes. The sequences between the cutting positions corresponding to the overhangs are indicated by altered coloring in the boxes. RES: Body Double Type IIS restriction endonuclease, its recognition site is represented by a green box, the sequences between its cleavage sites correspond to a Type IIP overhang are represented by the same orange box.

Genetic engineers' choices are frequently restricted to one or a few restriction endonucleases to clone a DNA fragment precisely to a specific position in a vector. The primary usefulness of employing restriction endonucleases as Body Doubles becomes evident when the problem arises of having a recognition site of the chosen restriction enzyme in the PCR fragment to be cloned. In such cases, the restriction enzyme that should be used for generating the overhang on the PCR fragment is simply substituted in the cloning strategy with a BD ENase that is able to generate the same overhang without cutting the PCR fragment at the pre-existing internal recognition site of the “*original*” Type IIP enzyme. This is achieved by incorporating (I) the recognition site of a Type IIS ENase that generates the same type of protruding ends (in respect to the 3′ or 5′ ends and the length of the overhang) as the original enzyme and (II) a sequence at the cutting position of the Type IIS enzyme - that is designed in such a way that the overhang after the digestion will be identical with the overhang of the original Type IIP enzyme - into the PCR primer. Thus, digestion with the Body Double enzyme will generate overhangs on the PCR fragment that can be ligated to the acceptor vector opened with the original Type IIP restriction enzyme without the necessity of any modifications to the vector (Figure 1).

This application of Body Double enzymes considerably extends the probability of finding appropriate ENases for a particular cloning task. We regularly use this approach in our laboratory and present several examples where without the application of Body Doubles for Type IIP restriction enzymes only a more cumbersome cloning strategy would be possible that might for instance involve any one of the following: using partial digestion of the PCR product, mutating the internal restriction site in the insert, introducing a new restriction site to the vector by a linker or by mutagenesis or ligating an adapter to the vector.

**Figure 2:**
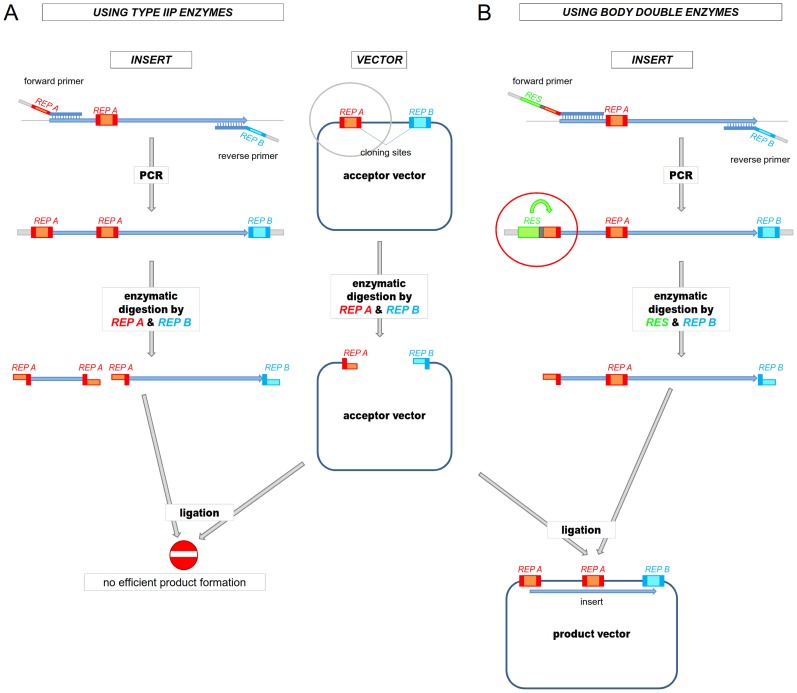
Schematic representation of a cloning strategy using a Body Double ENase. The use of a Type IIP ENase is not feasible for creating the desired overhang at the end of a PCR fragment due to the presence of an internal restriction site of the Type IIP ENase in the PCR fragment. **A.**) Digestion by the Type IIP restriction endonuclease cleaves the PCR fragment into two parts. Alternative strategy needs to be exploited. **B.**) Digestion by a Body Double Type IIS restriction ENase creates the appropriate overhangs on the PCR fragment identical to the overhangs that would be generated by the Type IIP ENase. The PCR fragment remains intact. REP A and B: Type IIP restriction enzymes, their recognition sites are represented by red and blue boxes, respectively. The sequences between the cutting positions corresponding to the overhangs are indicated by altered coloring in the boxes. RES: Body Double Type IIS restriction endonuclease, its recognition site is represented by a green box. The sequences between its cleavage sites corresponding to a Type IIP overhang are represented by an orange box. The flanking bases with arbitrary sequence needed for efficient enzymatic digestion of the PCR fragment are marked by light grey.

To exemplify the kind of experiments where the use of BD enzymes is advantageous we present a scheme (Figure 1 and 2) and describe cloning reactions involved: NdeI (CA′TA_TG) and NcoI (C′CATG_G) are the two Type IIP restriction ENases that can facilitate the easy insertion of a sequence coding for a peptide or protein following the ATG triplet in the vector without the addition of extra amino acids. For this reason NcoI and NdeI are frequently used in the cloning site of expression vectors and hence the presence of an NdeI or NcoI site(s) in the cDNA of proteins is more likely to interfere with certain cloning strategies than in the case of other Type IIP enzymes. Here we demonstrate the use of a Body Double for NcoI to clone the cDNA of ten different polypeptides in various different constructs.

The cloning site of the pTXB3 vector contains a unique NcoI site that allows seamless incorporation of a fragment immediately following the first ATG triplet. Unfortunately, many of the DNA fragments we planned to clone to this vector, such as the cDNA of mCherry, the mouse Prion protein (mPrP) and its fragments and mutant variants contain NcoI sites. We use BsmBI as a Body Double of NcoI to digest the PCR fragments and generate NcoI overhangs (Figure 1B). The other end of the PCR fragments is digested with SapI and the fragments are cloned between the NcoI and the SapI sites of the pTXB3 vector. Note, that the SapI enzyme is also a Type IIS ENase, but it is used here for the precise insertion of the fragment not as a Body Double restriction endonuclease.

**Figure 3:**
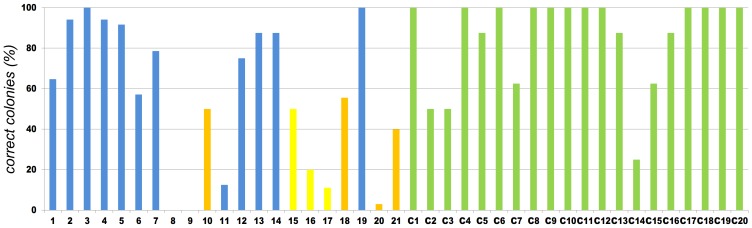
Cloning efficiency of constructs made by using BD (1–21) or Type IIP enzymes (C1–C20). The number of colonies containing the correct constructs was counted for each experiment and the percentage of those containing the correct constructs as a proportion of all tested clones was calculated. Experiments 1 to 21 were made using BD enzymes (blue, orange, yellow), and experiments C1 to C20 were made using Type IIP enzymes with background clearing (green). There was no possibility of decreasing the background in the experiments represented by orange columns. The constructs represented by yellow columns were made by inserting multiple inserts into the vector in a single ligation step. These experiments were not included in the calculation of the average BD efficiency. The efficiencies are 84,38% for the BD constructs and 83,80% for the Type IIP constructs. There is no data for samples 8 and 9.

Table 1 shows some of our recent cloning experiments where a Body Double enzyme has been employed to facilitate the cloning of a PCR fragment (Table 1 and Supplementary Table S1, cases 1–18). To demonstrate the feasibility of our approach, the cloning efficiency of 20 such PCR cloning experiments using Body Double enzymes is compared to 20 randomly selected cloning experiments carried out in our lab where was no need to use Body Double for the Type IIP enzyme applied. The number of correct clones as a percentage of all clones tested by restriction digestion are depicted in Figure 3. The cloning efficiencies using Type IIS enzymes were within the same range obtained when using Type IIP ENases. The average cloning efficiencies were comparable with or without using Body Double enzymes for constructs that were made by similar cloning strategies (Figure 3).

**Table 1:** The primers used in this publication that contain Body Double restriction enzyme sites.

oligo	sequence
1	agatcg*cgtctc*c**′**CATGgtgagcaagggcgaggag
2	ctgatg*cgtctc*c**′**CATG**a**aaaagcggccaaagcctgg
3	ctgatg*cgtctc*c**′**CATG**a**aaaagcggccaaagcctgg
4	taaggc*cgtctc*c**′**CATG**c**aaggaggaggtacccataatcagtg
5	taaggc*cgtctc*c**′**CATG**c**aaggaggaggtacccataatcagtg
6	ctgatg*cgtctc*c**′**CATG**a**aaaagcggccaaagcctgg
7	ctgatg*cgtctc*c**′**CATG**a**aaaagcggccaaagcctgg
8	gctata*cgtctcc* **′**CATG**a**agggcggccgcgga
9	gctata*cgtctcc* **′**CATG**a**agggcggccgcgga
10	aaaaa**′**TGCAg*gagacg*ctatcccacgatcaggaag
11	aaggag*cgtctc*c**′**CATG**a**aaaagcggccaaag
12	aca*cgtctc*g**′**GTACcttacaatttacgcgttaagatac
13	ccg*cgtctc*a**′**GTACccgtggtggtgatgatggtgc
14	gagaaa*cgtct*ca**′**AGCT**c**taggatcttctcccgtcgta
15a	aaaaaa*cgtctc*g**′**GGCC**aa**ggaggagctcaaggc
15b	aaaaaa*cgtctc*g**′**GATCctctctgcgcgtggcggctg
16	aaaaaa*cgtctc*g**′**GGCC**aa**ggaggagctcaaggc
17	aaaaaa*cgtctc*g**′**GGCC**aa**ggaggagctcaaggc
18a	atata*cgtctc*a**′**CCGGccgaattctaaggg
18b	atata*cgtctc*a**′**CTAGaggatcctaaggc
19a	gggggg**′**CCGG**a** *gagacg*cgcagatccaaaaaagaag
19b	ggggggg**′**CCGG**g** *gagacg*ttatctacctttctcttctt
20	ggggggg**′**CCGG**g** *gagacg*ttatctacctttctcttctt
21a	agcta*cgtctc*a**′**GTACgcgacgagaagaccac
21b	atcagatata*cgtctc*c**′**TCGAgcgcctc

The recognition sites of the enzymes are in *italic*; the overhangs generated are in CAPITALS. The cutting position is marked by a bold apostrophe. **Bold** and underline marks the bases which were altered in order to change the recognition sequence of the Type IIP ENase used.

### 2. Other possible uses of Body Double enzymes

Beside this major use of Body Double ENases described above, there are other unique situations where the employment of a BD enzyme for generating overhangs of Type IIP enzymes on a PCR fragment offers clear advantages. These applications require only small variations in the cloning strategies that employ a Body Double ENase as the following examples demonstrate.

#### 2.1 Using Body Doubles to remove the cloning site in the product vector after ligation

**Figure 4:**
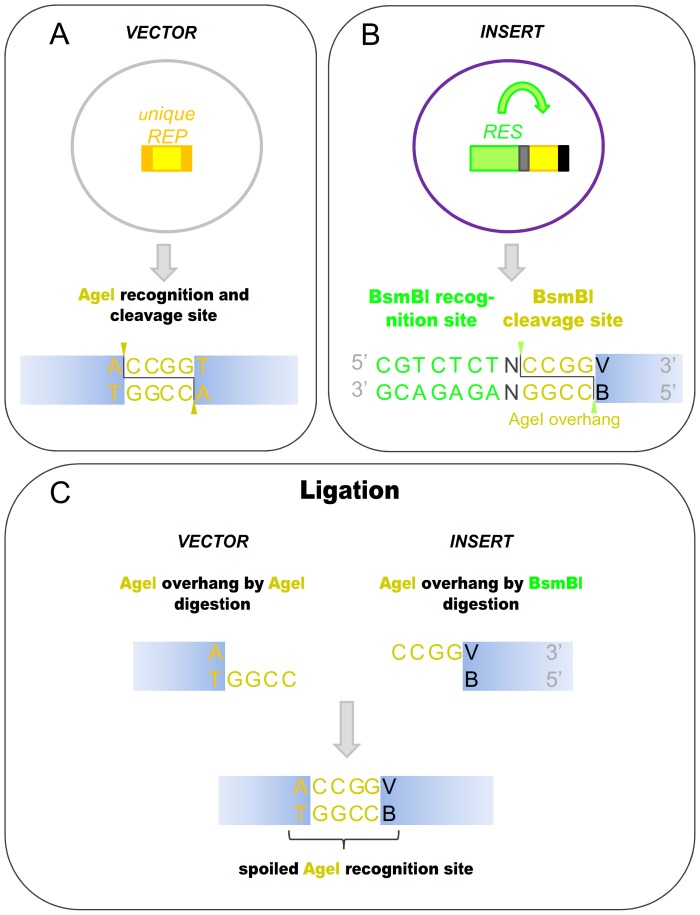
Ligation of AgeI overhangs generated by AgeI and its Body Double does not reconstitute the AgeI recognition site. **A.**) Sequence of AgeI recognition and cleavage site **B.**) Sequence of BsmBI recognition and cleavage site, the latter corresponding to an AgeI overhang **C.**) Ligation of the AgeI overhangs generated by different restriction enzymes does not reconstitute the AgeI site. REP: Type IIP restriction enzymes, their recognition sites are represented by orange boxes. The sequences between the cutting positions corresponding to the overhangs are indicated by altered coloring in the boxes. RES: Body Double Type IIS restriction endonuclease, its recognition site is represented by a green box, the sequences between its cleavage site correspond to a Type IIP overhang are represented by a yellow box. Arbitrary bases (N) are light grey, altered bases that destroy the AgeI recognition site are shown in black. V and B represent G,C or A and G,C,or T, respectively.

**Figure 5:**
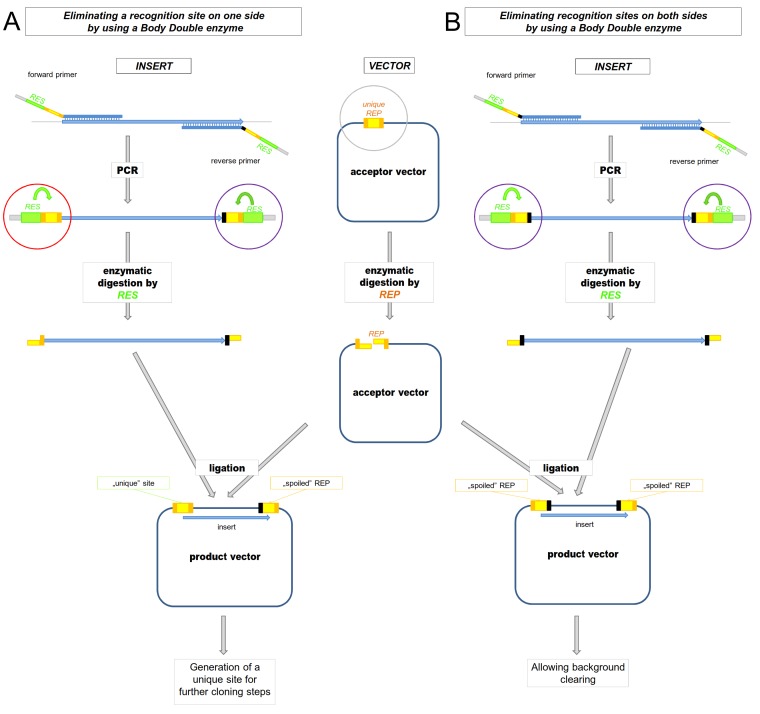
Schematic representation of a cloning strategy using a Body Double ENase when the Type IIP recognition sites in the vector are not reconstituted by the ligation at *A.)* one of the ends or *B.)* both ends of the insert. Digestion with a Body Double Type IIS restriction endonuclease creates the appropriate overhangs on the PCR fragment that are identical to the overhangs generated by the Type IIP ENase. **A.**) Only one of the REP restriction sites is reconstituted by the ligation at one end of the insert, and thus a unique REP site is formed in the vector. **B.**) The REP restriction sites are not reconstituted by the ligation at either ends of the insert. No REP site remains in the vector. REP: Type IIP restriction enzyme, its recognition site is represented by orange boxes. The sequences between the cutting positions corresponding to the overhangs are indicated by altered coloring in the boxes. RES: Body Double Type IIS restriction endonuclease, its recognition site is represented by green boxes. The flanking bases needed for efficient enzymatic digestion of the PCR fragment are marked in light grey.

One such useful application of Body Double digestion is when the cloning site(s) in the vector needs to be eliminated from the product vector. The Type IIP ENases generally have a recognition site that also includes bases flanking the cleavage site. Thus, when primers are designed to determine the specificity of a Body Double enzyme, it is possible to either keep or destroy the Type IIP recognition site in the final vector product by varying the sequence of the flanking parts without changing the sequence of the overhang (Figure 4). The absence of the site used for cloning may be demanded (i) by downstream steps in a particular cloning strategy or (ii) for decreasing/clearing the background (Figure 5).

**Figure 6:**
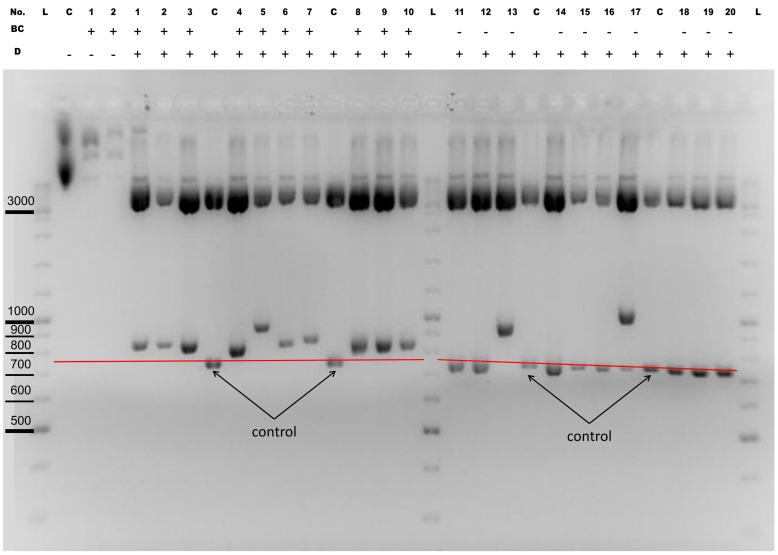
Efficiency of background clearing. 1.8% Agarose gel showing clones with (1–10) and without (11–20) background clearing (case 19). The plasmid DNA purified from colonies digested with NdeI for the presence of insert DNA. Background clearing was carried out before transforming the ligation reactions to competent *E. coli* bacteria by digesting (clones 1 to10) or without digesting (clones 11 to 20) the ligation reactions with the appropriate Type IIP enzyme (AgeI) used for cloning. The acceptor vector contains an AgeI site while the correct product vector does not. Thus, AgeI digestion linearized the circular acceptor vectors that contain no inserts thereby decreasing the empty vector background. After background clearing (1 to 10) none of the colonies contains an empty vector. In contrast, without background clearing 9 out of the 10 colonies have empty vectors. Some of the constructs apparently contained more than one insert. (No.: Numbers of the tested colonies, BC: background clearing, D: test digestion (expected fragments after NdeI digestion: 842, 4410; if multiple inserts were ligated, the length of the 842 fragment increases by ∼100 for each additional insert), C: control plasmid (expected fragments after NdeI digestion: 743, 4410), L: DNA ladder). The red line represents the mobility of the 743 bp NdeI fragment of the control vector.

(i) We faced the problem of needing to destroy the cloning site when we wanted to subclone a PCR fragment coding the nuclear localization signal of the Simian Vacuolating virus 40 (SV40) T-antigen between the endoplasmic reticulum-targeting signal sequence of mouse Shadoo protein (mSho) and the cDNA of EYFP (enhanced yellow fluorescent protein). Here, the Body Double restriction ENase is used to generate AgeI overhangs. The primer was designed in such a way that although the Body Double digestion of the PCR fragments generates AgeI overhangs, the subsequent ligation with the protruding ends of the vector generated by the *real* AgeI enzyme would not reconstitute the AgeI site. As the vector had been previously linearized only by AgeI at one position, cloning a fragment normally reconstitutes two AgeI sites flanking the insert. Here, destroying one of the AgeI sites leaves a unique AgeI site in the vector after ligation that could be used in subsequent cloning steps (Figure 5A, Supplementary Table S1, case 20).

(ii) Another situation is when the use of Body Double enzymes to eliminate the cloning site in a vector product facilitates a cloning strategy where the empty vector background can be decreased by a post-ligation re-digestion step with the Type IIP ENase used for cloning. This problem emerges, for example, when a PCR fragment needs to be cloned to a single cloning site. The difficulty with such a cloning strategy is that the elimination of the background by digestion of the ligation reaction with a restriction enzyme (that has a recognition site only in the acceptor vector but not in the final vector product) is not possible since there are no DNA sequences that can be found only in the original vector but not in the vector product. If a Body Double enzyme is used to create the appropriate overhangs on the ends of the PCR fragments, the cloning site in the vector product can be destroyed as a consequence of the ligation. As a result the background among the transformants caused by the (re-)circularized vectors not containing the PCR insert can be selectively removed (Figure 5B). We applied this strategy to clone a PCR fragment coding the nuclear localization signal of SV40 T-antigen between the cDNA of EYFP and the mouse Shadoo protein to an AgeI site (Figure 5B, Supplementary Table S1, case 14 and 19). The vector was linearized by AgeI, the PCR fragment by BsmBI to generate an AgeI overhang in such a way that the AgeI sites are destroyed after ligating the PCR fragment into the vector. To demonstrate the efficiency of this approach, the ligation reaction was used to transform competent bacteria after either performing or not performing a digestion with AgeI to clear the background. As a result of the digestion, the number of colonies on the plate decreased by an order of magnitude. 12-12 colonies were picked up and examined for the presence of the insert by purifying DNA from each clone and by restriction digestion. As expected from the relative number of colonies on the two plates after AgeI digestion none of the tested clones did not contain an insert. By contrast, among the undigested control samples, 10 out of the 12 clones contained only empty vectors (Figure 6).

#### 2.2 Using Body Double ENase for cloning to NcoI site without altering the second amino acid following the ATG in the protein sequence

**Figure 7:**
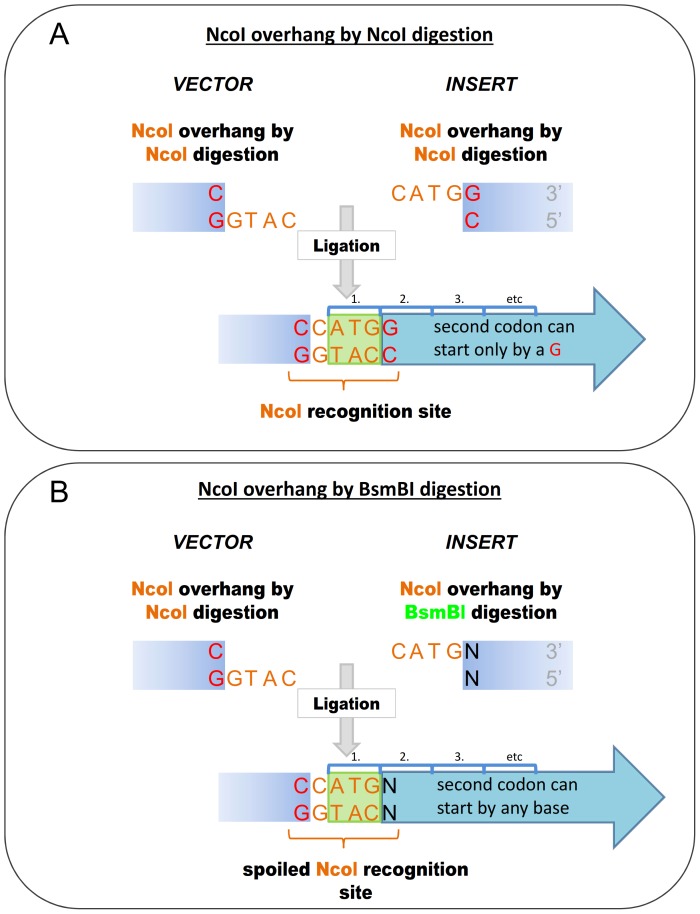
Schematic representation of a cloning strategy using a Body Double of NcoI to allow the coding of any amino acids following the starter methionine. The last G base in the NcoI recognition site is altered in order to freely choose the codon after the ATG. **A.**) Ligation of the NcoI overhangs (orange) of the vector and insert reconstitutes the original NcoI site, therefore the codon for the second amino acid following the starter methionine can only start by a G base. **B.**) Ligation of NcoI overhangs (orange) where the overhang of the insert is generated by a BD enzyme to allow the last base of the NcoI site to be freely selected. Thus, the codon for the second amino acid following the starter methionine is not determined by the cloning procedure.

Altering the recognition site by using a Body Double may be particularly advantageous in the case of NcoI. Whereas linearization of the vector with NdeI allows any amino acid to be coded for by the codon following the first ATG codon, using NcoI (its recognition and cleavage site C′CATG_G) the possible amino acids that can be coded for in the second position is limited to codons starting with a G. When a BD of NcoI is used, the base in the position of the 3′ final G in the original NcoI site can be altered at will, allowing the second amino acid coded for to be freely determined thus permitting the expression of polypeptides without introducing a mutation (Figure 7, Supplementary Table S1, cases 2–9 and 11).

#### 2.3 Using a single Body Double enzyme for two Type IIP ENase generating different overhangs on the two ends of a PCR fragment in a single digestion

An additional useful application of a Body Double enzyme is when the type of the protruding ends (with respect to the 3′ or 5′ ends and the length of the overhang) to be generated is identical at the two ends of a PCR fragment. Here a single Type IIS enzyme can be used as the Body Double of two different ENases at the same time. This strategy has the advantage that the digestion can be performed in a single step. Because PCR fragments are frequently digested O/N and because the two selected restriction enzymes frequently have no compatible buffer for co-digestion, this strategy may provide a considerable timesaving advantage (Supplementary Table S1, case 21).

### 3. Online Body Double Finder Program

To assist the process of finding Body Double enzymes, we created an online program (http://group.szbk.u-szeged.hu/welkergr/body_double/index.html) that facilitates the selection of suitable BD enzymes and the design of the appropriate primers (see the materials and methods section).

## Discussion

The general usefulness of our strategy derives from the combination of two elementary procedures:

The first is the use of a Type IIS restriction enzyme as a Body Double for a Type IIP enzyme i.e.: to generate palindromic overhangs using a Type IIS enzyme that are identical to the overhangs of a particular Type IIP restriction enzyme. We realise that there are also a few Type IIP enzymes that generate identical overhangs while having different recognition sites (e.g.: BamHI and BglII or NheI, XbaI, AvrII and SpeI). However, here, we are able to assign to a single Type IIS enzyme a wide variety of palindromic overhangs each of which is identical to that of a Type IIP enzyme. Type IIS ENases are generally exploited to generate non-palindromic overhangs for fragment assembly, facilitating directional cloning and/or creating unique junction sites [[7], [8], [9], [10], [11], [28] for review: [5]]. In their early work, Rebatchouk et al. constructed vectors where four ENases, three Type IIS and one Type IIP were oriented to cut at the same positions and generate the same non-palindromic sequence for *nucleic acids ordered assembly with directionality (NOMAD)* [27]. The general idea that Type IIS enzymes can serve as Body Doubles for Type IIP enzymes has already been put forward by us as a way to construct cloning sites in a vector without the need for eliminating the existing recognition sites in the vector backbone by mutagenesis [29]. However, since this publication only a few papers have made use of the idea of generating identical overhangs with a Type IIP enzyme [26], [32]. We think this is likely to be a consequence of the practical hurdles associated with its suggested implementation [26], [29].

The second procedure, and the one that makes these specificities readily available is that we employed the Body Double enzyme, the Type IIS ENase on a PCR fragment (as opposed to a vector) to generate Type IIP enzyme compatible overhangs. Thus, just by redesigning the flanking region of the PCR primer it is possible to switch from the use of a Type IIP enzyme to the use of its Type IIS Body Double. The absence of the need to use any special, pre-modified vectors for these cloning reactions makes our strategy a very useful general approach. Our present strategy also makes Type IIS ENases almost as useful as commonly-used Type IIP ENases for general cloning applications.

Many former approaches applying Type IIS restriction enzymes in cloning procedures required the careful engineering of the overhangs of the Type IIS enzyme in both the vector and the insert since the Type IIS enzymes would otherwise to generate non-compatible overhangs. This either restricts the use of Type IIS enzymes to sometimes very sophisticated but specific applications using specific vectors or makes their general use impractical due to the requirement for extra cloning steps [5], [7], [8], [9], [10], [11], [21], [24], [26], [27], [28], [29]. In contrast, when using a Type IIS ENase as a Body Double palindromic overhangs are generated on a PCR fragment that are compatible, and thus, suitable for joining with the overhangs generated by the “original” Type IIP ENase. Thus, whenever a Type IIP enzyme is used in a vector for cloning, its Body Doubles can also be used provided the insert is a PCR product.

A Type IIS enzyme can be frequently turned into a Body Double for several Type IIP enzymes that produce identical type of overhangs (in terms of 3′ or 5′ and the lengths of the protruding ends). The cleavage specificity of a BD enzyme can be switched among the existing cleavage specificities of the corresponding Type IIP ENases at the discretion of the investigator by the appropriate design of a PCR primer. Thus, they can be used like a Joker in cloning tasks.

For many of the Type IIP ENases, our approach, using the search program we have written, allows the identification of a number of Type IIS ENase counterparts producing identical overhangs although recognising different sequences. Thus, our strategy increases the versatility with which strategies for a particular cloning task can be devised. Once an ENase is found that can linearize the vector, the chance that a PCR fragment with compatible overhangs can be produced (without cutting inside of the PCR fragment) is greatly increased by using Body Double ENases.

**Figure 8:**
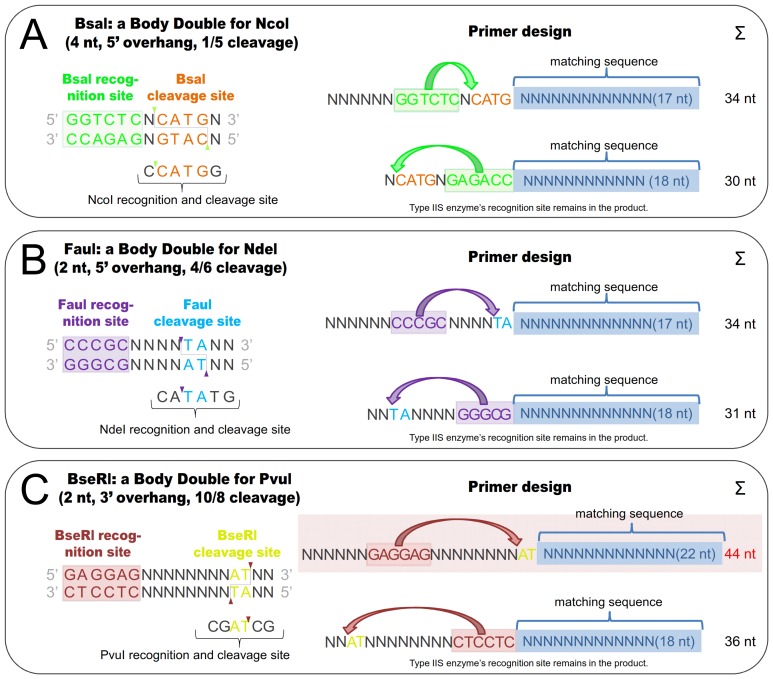
Layouts of generalized primers containing BD ENase sites useable to generate different type of overhangs and used in different orientations. These layouts are based on the outputs of the Body Double Finder online program. A primer containing the site of a RES generating **A.**) 4 nt, 5′ overhang **B.**) 2 nt, 5′ overhang and **C.**) 2 nt, 3′ overhang. To the left is the recognition sequence of BsaI, FauI and BseRI respectively (recognition site: green, purple and red letters; cleavage site: orange, blue and yellow letters respectively). Green, purple and red triangles mark where cutting occurs, and the lines show the overhangs generated. To the right are possible primer layouts. In the primers shown uppermost the RES removes its recognition site from the PCR fragment while in the other primer (below) the recognition site of the Body Double RES remains in the insert after digestion. In the first case the oligo primer must contain an oligonucleotide tail in order to facilitate the cleavage by the restriction enzyme at the very edge of the primer. Such an extra tail is not required in the second case since the cleavage site already flanks the recognition site. Increasing the distance between the recognition and cleavage sites results in longer primers, therefore in ***C.*** *)* only one orientation of the Body Double RES site can be accommodated in a PCR primer of the suggested length.

When Type IIS restriction enzymes are employed as Body Doubles they offer many but not all the advantages of both Type IIP and IIS enzymes. Thus, some features of the Type IIS enzymes that have been demonstrated before in specific applications [5] can now be explored without the necessity of extra steps for pre-modifying the vector. One of these advantages is that by varying the orientation of the recognition site of the Type IIS enzyme in the sequence of the primer (i.e.: by positioning the cleavage site upstream or downstream of the recognition site) it is possible to ensure the presence (at one or both sides of the insert for subsequent cloning tasks) or the absence (for efficient removing of the empty vector background by the Type IIS ENase) of the recognition site of the used Type IIS enzyme in the vector product (Figure 3, primer design). However, it should be noted, that when the distance between the recognition and cleavage sites is longer (10–12 bases) only one orientation can be accommodated in primers of the length we used in these experiments: if the cleavage site is situated at the 5′ end of the primer (Figure 8). Although using a longer primer tail is feasible, we suspect that the efficiency of the PCR reaction may decrease with increasing non-matching primer length.

A possible further development of the applications used in this paper is the use of nicking ENases as Body Doubles for Type IIP enzymes. It is expected that the cloning strategy would be much less sensitive to the presence of internal recognition site of the nicking enzyme in the PCR fragment than if a Type IIS ENase was used. However nicking enzymes could be used in one orientation only if their recognition site is to remain in the product vector.

In Table 2 we list the Type IIS restriction enzymes that are suitable for being used as Body Doubles. Some Type IIP ENases recognising interrupted palindromes may be also suitable as Body Doubles, provided that they generate overhangs with arbitrary sequences. The basic criteria that must be fulfilled to qualify a Type IIS enzyme as a BD are: (I) the Type IIS restriction ENase needs to cut in a predictable manner, i.e.: at all sequences that are identical with its recognition site but at no sequences with even a one base alteration from it. (II) In addition, the cutting always needs to occur precisely at a given distance from the recognition site - characteristic for the particular Type IIS restriction ENase. (III) It has to generate protruding ends that can be efficiently religated. (IV) The protruding ends should be of the same type (with respect to 3′ and 5′ and the length of the overhangs) as those that a Type IIP restriction ENase generates. This usually means 2 or 4 base-long protruding ends in the case of both the 3′ and 5′ overhangs generated by Type IIP restriction enzymes.

**Table 2:** List of ENases usable as Body Doubles.

2 nt overhang	
Enzyme name	Recognition site and cleavage position
**3′ overhang**	
BtsCI, BstF5I, BseGI	GGATGNN′
BsrDI, Bse3DI, BseMI	GCAATGNN′
BtsI	GCAGTGNN′
BspCNI, BseMII	CTCAGNNNNNNNNNN′
BseRI	GAGGAGNNNNNNNNNN′
EciI	GGCGGANNNNNNNNNNN′
TaqII^1^	GACCGANNNNNNNNNNN′
	CACCCANNNNNNNNNNN′
TsoI^1^^,^ 2	TARCCANNNNNNNNNNN′
TspDTI^1^	ATGAANNNNNNNNNNN′
TspGWI^1^	ACGGANNNNNNNNNNN′
DrdI, AasI^3^	GACNNNN′NNGTC
**5′ overhang**	
FauI, SmuI	CCCGC′NN
Hpy188III^3^	TC′NNGA

4 nt overhang	
Enzyme name	Recognition site and cleavage position
**5′ overhangs**	
BsmAI, Alw26I, BcoDI	GTCTCN′NNNN
BsaI, Bso31I, Eco31I	GGTCTCN′NNNN
BsmBI, Esp3I	CGTCTCN′NNNN
BbsI, BpuAI, BstV2I, BpiI	GAAGACNN′NNNN
AarI	CACCTGCNNNN′NNNN
BspMI,BfuAI, Acc36I, BveI	ACCTGCNNNN′NNNN
SfaNI, LweI	GCATCNNNN′NNNN
BseXI, BbvI, Lsp1109I, BstV1I	GCAGCNNNNNNNN′NNNN
SgeI	m5CNNGNNNNNNNNN′NNNN
FokI	GGATGNNNNNNNNN′NNNN
**3′ overhang**	
BstXI^3^	CCANNNNN′NTGG

Commercially available enzymes suitable for being Body Doubles of a Type IIP ENase are grouped according the type of the generated overhangs. ENases of which recognition and cleavage sites need more than 18 bases to be accommodated in a primer are not listed. Isoschizomers are listed in the same row, separated by commas.

1 ) No data about religation efficiency.

2 ) No data about cutting predictability.

3 ) Type IIP ENase with interrupted recognition site.

In order for a Body Double enzyme to be useful in practice, one additional criterion needs to be met: the distance between the recognition and cutting sites should not be too long, so that it can be accommodated in a PCR primer. When a Type IIP restriction site is inserted into the 5′ end of a primer, some 12 extra non-matching bases need to be built into the primer; generally 6 bases for the recognition site of the Type IIP restriction enzyme and an additional 6 bases depending on the particular enzyme used to ensure efficient cleavage at the end of the DNA fragment. When a Type IIS enzyme is built to the end of the primer we inserted another up to 6 additional bases (Table 1 and Supplementary Table S1) as the cleavage and recognition sites are separated from each other in the case of Type IIS enzymes. These extra bases seem to be sufficient to accommodate both sites of many of the Type IIS enzyme that are suitable for being used as BD enzymes. We routinely use this approach in our laboratory (see the 21 cases that are presented in Table 1 and Supplementary Table S1) and have not observed any problem with the efficiency of the PCR due to the longer (18-base-long) non-matching region at the end of the primers.

### Supplementary Data Statement

Supplementary Table S1 is available online, including Supplementary References [30], [31], [33].

## Supporting Information

Table S1 **Summarizing list of the used PCR primers, ENases, vectors and reaction conditions.** Primers: Digested overhangs by the BD enzymes are marked by CAPITAL LETTERS, the recognition sites of the BD enzymes are marked by *italic*. We generally use BsmBI as a BD enzyme as it cuts very close to its recognition site (which results in relatively short primers). Annealing temperature: two different degrees are shown when the temperature was raised after five cycles. Number of cycles: the temperature was raised after five consecutive cycles when needed. The pSB plasmid was a kind gift of Z. Ivics and Zs. Izsvák [30]. The pACS-KDEL-SS^IL2^-SBP-_E_GFP-GPI plasmid was a kind gift of F. Perez [31]. His: histidine (6×, 10×), _hM_GFP: monster green fluorescent protein, inz: inzulator, mPrP: mouse Prion protein (wt: wild type), _E_YFP: enhanced yellow fluorescent protein (ML: first metionin is mutated to leucin to avoid the expression of a signal peptide-less protein, Nyeste and Welker, unpublished results), cys: cysteine, GPI: signal sequence of the glycophosphatidylinositol phosphate anchor, SS: ER-targeting signal sequence (the signal sequences usually comes from the protein whose core sequence is incorporated in the plasmid construct, otherwise it is marked), mSho: mouse Shadoo protein, IL2: interleukine-2, KDEL: endoplasmic reticulum retention signal, SBP: streptavidin binding peptide
(PDF)
