# Supplementary material for: Restriction Enzyme Body Doubles and PCR Cloning: On the General Use of Type IIS Restriction Enzymes for Cloning
Source: PLoS One. 2014 Mar 11;9(3):e90896. doi: 10.1371/journal.pone.0090896 (PMC3949710; doi:10.1371/journal.pone.0090896)
Supplement: Table S1 — Summarizing list of the used PCR primers, ENases, vectors and reaction conditions. Primers: Digested overhangs by the BD enzymes are marked by CAPITAL LETTERS, the recognition sites of the BD enzymes are marked by italic. We generally use BsmBI as a BD enzyme as it cuts very close to its recognition site (which results in relatively short primers). Annealing temperature: two different degrees are shown when the temperature was raised after five cycles. Number of cycles: the temperature was raised after five consecutive cycles when needed. The pSB plasmid was a kind gift of Z. Ivics and Zs. Izsvák [30]. The pACS-KDEL-SSIL2-SBP-EGFP-GPI plasmid was a kind gift of F. Perez [31]. His: histidine (6×, 10×), hMGFP: monster green fluorescent protein, inz: inzulator, mPrP: mouse Prion protein (wt: wild type), EYFP: enhanced yellow fluorescent protein (ML: first metionin is mutated to leucin to avoid the expression of a signal peptide-less protein, Nyeste and Welker, unpublished results), cys: cysteine, GPI: signal sequence of the glycophosphatidylinositol phosphate anchor, SS: ER-targeting signal sequence (the signal sequences usually comes from the protein whose core sequence is incorporated in the plasmid construct, otherwise it is marked), mSho: mouse Shadoo protein, IL2: interleukine-2, KDEL: endoplasmic reticulum retention signal, SBP: streptavidin binding peptide (PDF) [file pone.0090896.s001.pdf]

No.	insert	forward primer	reverse primer	Body double for?	acceptor vector	annealing temperature	number of cycles
**1**	$_{m}$Cherry	agatcg*cgtctc*cCATGgtgagcaagggcgaggag	cgagacgctcttccgcacttgtacagctcgtcc	NcoI	pTXB3^{10xHis}	58°C → 72°C	5 + 30
**2**	mPrP^{wt}	ctgatg*cgtctc*cCATGaaaaagcggccaaagcctgg	aaaagcgctcttctgcaggatcttctcccgtcg	NcoI	pTXB3	58°C → 72°C	5 + 30
**3**	mPrP$^{wt\ 3f4}$	ctgatg*cgtctc*cCATGaaaaagcggccaaagcctgg	aaaagcgctcttctgcaggatcttctcccgtcg	NcoI	pTXB3	58°C → 72°C	5 + 30
**4**	mPrP (90-232)	taaggc*cgtctc*cCATGcaaggaggaggtacccataatcagtg	gagcgcgctcttcagcagctactggatcttctcccgtcgtaataggc	NcoI	pTXB3^{10xHis}	64°C → 72°C	5 + 30
**5**	mPrP (90-232)^{3f4}	taaggc*cgtctc*cCATGcaaggaggaggtacccataatcagtg	gagcgcgctcttcagcagctactggatcttctcccgtcgtaataggc	NcoI	pTXB3^{10xHis}	64°C → 72°C	5 + 30
**6**	mPrP (23-177)^{3f4}	ctgatg*cgtctc*cCATGaaaaagcggccaaagcctgg	atggtggctcttccgcagtcgtgcacgaagttgttctgg	NcoI	pTXB3	58°C → 72°C	5 + 30
**7**	mPrP (23-212)^{3f4}	ctgatg*cgtctc*cCATGaaaaagcggccaaagcctgg	tggtacgctcttccgcacatctgctccaccacgcgctcc	NcoI	pTXB3	58°C → 72°C	5 + 30
**8**	mSho	gctata*cgtctc*cCATGaagggcggccgcgga	agacaagctcttctgcagtgcacggaccctgagcc	NcoI	pTXB3^{10xHis}	63°C → 72°C	5 + 30
**9**	mSho-linker	gctata*cgtctc*cCATGaagggcggccgcgga	ctatgcgctcttctgcagccggaagtccaggcccag	NcoI	pTXB3^{10xHis}	63°C → 72°C	5 + 30
**10**	mPrP^{wt}	aaaaaaccgggagacgatggcgaaccttggctactg	aaaaaTGCAg*gagacg*ctatcccacgatcaggaag	PstI	psiSTRIKE-$_{hM}$GFP	60°C	30
**11**	mPrP^{wt}-$_{m}$Cherry	aaggag*cgtctc*cCATGaaaaagcggccaaag	cggatcaagcttcaacaatggtggtgatgatg	NcoI	pTrcHisA	55°C → 65°C	5 + 35

**12**	mPrP^{wt}-$_{E}$YFP-GPI	tttccctcatgaacgttatcccctgattctgtg	aca*cgtctc*gGTACcttacaatttacgcgttaagatac	BsrGI	pSB^{inz} [30]	55°C → 65°C	5 + 35
**13**	mPrP(29-135)-$_{m}$Cherry	ggaggatggaacaccggtggaagtcgataccc	ccg*cgtctc*aGTACccgtggtggtgatgatggtgc	BsrGI	pSB^{inz}-mPrP^{wt}-$_{E}$YFP-GPI	55°C → 65°C	5 + 35
**14**	mPrP^{wt}	cgcgatcacatggtcctcaaggagttcgtgaccgc	gagaaa*cgtctc*aAGCTctaggatcttctcccgtcgta	HindIII	pRSETb-GFP$^{6xHis\ cys}$	55°C → 65°C	5 + 35
**15**	mSho^{RQ}	aaaaaa*cgtctc*gGGCCaaggaggagctcaaggc	gggggggatccgtattgtggtgctggttg	NotI	pSS-mSho-$_{E}$YFP-GPI [33]	57°C	40
		aaaaaa*cgtctc*gGATCctctctgcgcgtggcggctg	cccccgaccggtaaggaagtccaggcccag	BamHI		67°C → 70°C	5 + 35
**16**	mSho^{RQ}	aaaaaa*cgtctc*gGGCCaaggaggagctcaaggc	cccgaaggatcctaaggccgaagcagttctag	NotI	pSS-$_{E}$YFP^{ML}-mSho-GPI [33]	61°C → 70°C	5 + 35
**17**	mSho^{RQ}	aaaaaa*cgtctc*gGGCCaaggaggagctcaaggc	cccgaaggatcctaaggccgaagcagttctag	NotI	p$_{E}$YFP-mSho-GPI [33]	66°C → 70°C	5 + 35
**18**	mSho-GPI	atata*cgtctc*aCCGGccgaattctaaggg	atata*cgtctc*aCTAGaggatcctaaggc	NgoMIV, XbaI	pACS-KDEL-SS^{IL2}-SBP-$_{E}$GFP-GPI [31]	49°C → 70°C	5 + 35
**19**	NLS^{x3SV40}	ggggggCCGGa*gagacg*gcagatccaaaaaaagaag	gggggggCCGGg*gagacg*ttatctacctttctcttctt	AgeI	pSS-mSho-$_{E}$YFP-GPI [33]	48°C	40
**20**	NLS^{x3SV40}	ggggggaccggtcgcagatccaaaaaaagaag	gggggggCCGGg*gagacg*ttatctacctttctcttctt	AgeI	pSS-$_{E}$YFP^{ML}-GPI [33]	48°C	40

**21**	SBP-mSho (25-32)	agcta*cgtctc*aGTACgcgacgagaagaccac	atcagatata*cgtctc*cTCGAgcgcctc	Pfl23II, XhoI	pACS-KDEL-SS^{IL2}-SBP-$_{E}$GFP-mSho-GPI	50°C → 67°C	5 + 35
